# Supplementary material for: PD-L1 Expression in High-Risk Early-Stage Colorectal Cancer—Its Clinical and Biological Significance in Immune Microenvironment
Source: Int J Mol Sci. 2022 Oct 31;23(21):13277. doi: 10.3390/ijms232113277 (PMC9655444; doi:10.3390/ijms232113277)
Supplement: Supplementary file 1 [file ijms-23-13277-s001.zip › ijms-1966729-supplementary.pdf]

Supplementary Table S1. Clinical characteristics of the 184 early-stage CRC patients from the TCGA database.

Characteristics	No. of cases (%)
Total patients	184
Age, median (range) — yr	64 (31–90)
Gender	
Male	94 (51)
Female	90 (49)
Race	
White	140 (76)
Black or African American	31 (16.8)
Asian	12 (6.5)
American Indian or Alaska Native	1 (0.5)
Primary tumor location	
Colon	143 (77.7)
Rectum	41 (22.3)
TMN stage	
II	99 (53.8)
III	85 (46.2)

Supplementary Table S2. List of statistically significant enriched pathways in the high CD274 cluster, analyzed by GSEA.

	Gene Sets	Size	ES	NES	p-value	FDR q-value
1	ALLOGRAFT_REJECTION	183	0.71	2.34	0.000	0.000
2	IL6_JAK_STAT3_SIGNALING	84	0.74	2.31	0.000	0.000
3	INFLAMMATORY_RESPONSE	193	0.71	2.25	0.000	0.000
4	INTERFERON_GAMMA_RESPONSE	191	0.72	2.23	0.000	0.000
5	IL2_STAT5_SIGNALING	189	0.55	2.22	0.000	0.000
6	TNFA_SIGNALING_VIA_NFKB	194	0.66	2.15	0.000	0.001
7	COMPLEMENT	189	0.59	2.15	0.000	0.001
8	KRAS_SIGNALING_UP	182	0.55	2.10	0.000	0.001
9	INTERFERON_ALPHA_RESPONSE	91	0.7	2.01	0.004	0.004
10	ANGIOGENESIS	35	0.62	1.97	0.000	0.005
11	APOPTOSIS	150	0.44	1.84	0.000	0.021
12	HYPOXIA	182	0.43	1.72	0.021	0.048
13	UV_RESPONSE_DN	135	0.48	1.68	0.020	0.062
14	COAGULATION	122	0.42	1.61	0.031	0.091
15	HEME_METABOLISM	179	0.32	1.43	0.031	0.189

Supplementary Table S3. Correlation of CD274 with CD3G and CD68 in early-stage CRCs from TCGA database

Correlation between	r*	P value
CD274 and *CD3G*	0.720	<0.001
CD274 and *CD68*	0.555	<0.001

*Spearman correlation

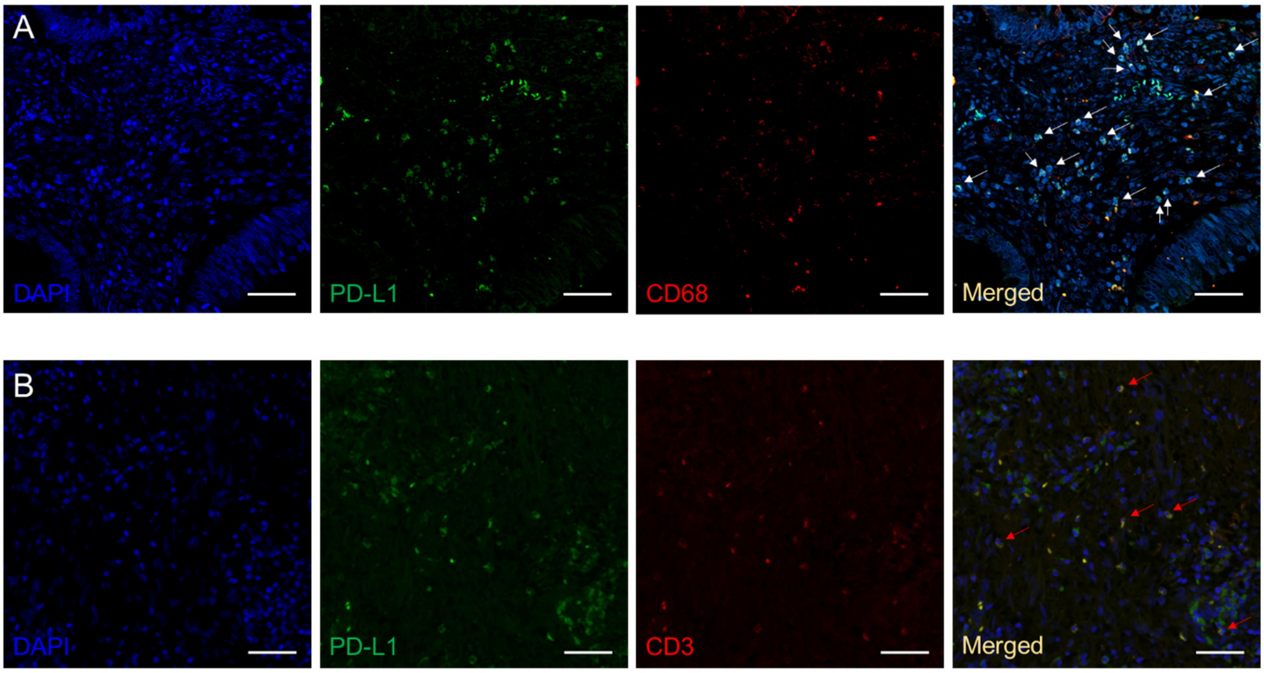

Supplementary Figure S1. PD-L1, CD68 and CD3 expression of immune cells in the representative case with high CPS. Representative immunofluorescent images of PD-L1 expression co-stained with anti CD68 antibodies (A) and anti CD3 antibodies (B) are shown. The white and red arrows indicate the colocalized foci of PD-L1 and CD68 (for macrophages) and PD-L1 and CD3 (for T lymphocytes), respectively. Scale car, 50 μm.
